# DNA State Influences the Uptake and Persistence of Environmental DNA by Marine Sponge Natural Samplers

**DOI:** 10.1002/ece3.70919

**Published:** 2025-05-23

**Authors:** Rosalie Dowell, Jamie Craggs, Catherine Head, Chris Yesson, Emma Ransome

**Affiliations:** ^1^ Institute of Zoology Zoological Society of London London UK; ^2^ Georgina Mace Centre for the Living Planet, Department of Life Sciences Imperial College London Ascot UK; ^3^ Horniman Museum and Gardens London UK

**Keywords:** degradation, DNA state, environmental DNA, natural sampler DNA, qPCR

## Abstract

Marine sponges as natural samplers of environmental DNA (eDNA) are receiving growing attention as an untapped source of biodiversity data. However, little is known about the state of DNA (e.g., cellular or extracellular) that is captured by these samples and how this compares to conventional aquatic eDNA samples. Here, we present an artificial spiking experiment where DNA in cellular and extracellular states was added into tanks containing two sponge species. Aquatic eDNA samples and sponge natural sampler DNA (nsDNA) samples were collected over 7 days and DNA from the two states was quantified in each sample using quantitative real‐time PCR (qPCR). We found that there was a significant interaction between DNA state and sample type (eDNA and nsDNA), with lower detection and concentration of extracellular DNA, compared to cellular DNA, found in nsDNA samples. We also found that detection rate and concentration of DNA were significantly lower in nsDNA than in eDNA overall. During methodological testing, PCR inhibition was observed in both sponge species; this was prohibitive in one of the species. Further work to investigate the degree of PCR inhibition during nsDNA metabarcoding is important to understand its impact on the communities resolved using nsDNA methods. *Synthesis and applications*. We show that nsDNA may originate from a subset of the DNA present in environmental media, potentially providing a more stable picture of local communities. Natural samplers provide a promising option for hard‐to‐reach environments and for retrieving biodiversity data from archived samples; however, further work and optimization are required to understand what is and is not well represented by this sample type compared to widely applied aquatic eDNA approaches.

## Graphical abstract

We present results from a mesocosm experiment testing the utility of marine sponges as natural samplers of eDNA and show the effect of DNA state on the capture and persistence of genetic material. We find a significant interaction between DNA state and sample type, with lower detection and concentration of extracellular DNA, compared to cellular DNA, found in nsDNA samples. This indicates nsDNA communities originate from a subset of DNA available in the environment.

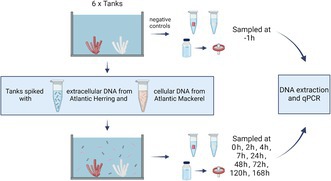

## 1 Introduction

In the marine environment, environmental DNA (eDNA) metabarcoding has been shown to facilitate noninvasive, cost‐effective, large‐scale, and multi‐taxa surveys that do not directly rely on taxonomic experts (Stat et al. 2017; Zhang et al. 2020). Samples of environmental media, such as seawater, are collected and processed to isolate DNA, which can be amplified using universal PCR assays and sequenced to target taxonomic groups of interest. A key strength of this method is its versatility, with many applications in a range of aquatic and terrestrial environments, and its ability to target organisms across the tree‐of‐life, producing large data sets, which are available for varied analyses (Chavez et al. 2021). However, as with the introduction of any novel method, eDNA methods can present challenges for their users, often requiring a complicated workflow of field, laboratory, and bioinformatic processes. Concerns have been raised around false positives and negatives that can be introduced by sampling regimes and contamination during processing, such as water filtering (Sepulveda, Hutchings, et al. 2020). Samples are also often collected at a single point in time due to logistical constraints, but recent studies show that communities detected by aquatic eDNA samples may be subject to short‐term variability, representing temporal changes in animal movement, behavior, and environmental conditions (Jensen et al. 2022; Ely et al. 2021; Dowell et al. 2024). To minimize these impacts, it is recommended to increase both the volume of samples collected (Bessey et al. 2020; Stauffer et al. 2021) and the sampling effort (Stauffer et al. 2021; Ely et al. 2021) to better resolve the spatial and temporal resolution of the communities detected by eDNA.

To tackle some of the challenges associated with water filtration and DNA isolation from environmental samples, there has been considerable investment in developing high‐tech and hands‐off solutions (e.g., passive samplers (Chen et al. 2022) and automated underwater vehicles (Hendricks et al. 2023)). While passive samplers such as the “metaprobe” (Maiello et al. 2022) and the HAp sampler (Verdier et al. 2022) show promising results, often recovering communities comparable to actively filtered water samples (Bessey et al. 2021; Verdier et al. 2022). However, many automated samplers are still prohibitively expensive, preventing their widespread application (Sepulveda, Birch et al. 2020; Formel et al. 2021). Recent studies have also highlighted a simpler alternative: the use of marine organisms, such as sponges (Porifera), as natural samplers of eDNA (nsDNA) (Mariani et al. 2019). Sponges are considered some of the most efficient natural water filters on Earth (Morganti et al. 2019; Kahn et al. 2015). Water is drawn into the internal vascularized canal system through external apertures (Godefroy et al. 2019), with water movement then primarily driven by negative pressure produced via the movement of flagellated choanocytes in internal chambers, resulting in the capture and concentrating of particulate matter (including eDNA) in their tissues (Wehrl, Steinert, and Hentschel 2007).

Mariani et al. (2019) first showcased this method, resolving a total of 31 metazoan taxa from nsDNA samples and describing distinct communities from the Antarctic and Mediterranean ecoregions. Subsequently, sequencing of archived sponges has successfully been used to characterize fish and eukaryotic communities in tropical, temperate, and polar sites (Turon, Angulo‐Preckler, et al. 2020; Neave et al. 2023; Jeunen, Lamare, et al. 2023; Cai et al. 2024). Experiments conducted by Cai et al. (2022) and Harper et al. (2023) have further helped refine nsDNA methods and investigated the detection and persistence of mock community DNA in marine sponges. The potential advantages provided by natural samplers in remote and logistically challenging sites have also been clearly highlighted by applications in the Southern Ocean (Jeunen, Miles, et al. 2023) and deep‐sea fisheries (Brodnicke et al. 2023).

Sponges, as natural samplers, offer many advantages for the sampling of environmental DNA: they are ubiquitous throughout the marine realm (van Soest et al. 2012), and due to regenerative properties, offer a nonfatal sampling method if sampled appropriately (Ereskovsky et al. 2021). As with passive sampling, the removal of filtering requirements also increases the speed and simplicity of sampling protocols. However, although applications of nsDNA for biodiversity assessment are increasing, many aspects of the ecology of nsDNA remain relatively untested. There are many factors, which may affect a sponge's filtering capability and the concentration and persistence of DNA within its tissues. The pumping rate of sponges is variable and has been shown to correlate with the density of choanocyte chambers (Massaro et al. 2012) and to oscillate over the diurnal cycle (Strehlow et al. 2016). Sponges are also often categorized into those with low microbial abundance (LMA) and high microbial abundance (HMA) (Moitinho‐Silva et al. 2017), which have been shown to occupy different trophic niches, and capture and utilize plankton and particulate matter in different capacities (Morganti et al. 2017). The impact of microbial activity on the degradation of DNA has been well‐documented in eDNA studies (Joseph et al. 2022; Saito and Doi 2021), and these attributes appear to make LMA sponges more appropriate candidates as natural samplers (Cai et al. 2022; Brodnicke et al. 2023). Weisz, Lindquist, and Martens (2008) found that HMA sponges also exhibit significantly lower pumping rates, a further potential reason for greater detection success when using LMA sponges. Another important consideration is the production of bioactive metabolites by sponges and their associated microbes, that are likely to inhibit enzymatic reactions, including those important for processing eDNA, such as PCR (Vargas et al. 2012; Harper et al. 2023).

In aquatic ecosystems, significant efforts have been made to understand how eDNA interacts with and persists in the environment, including its release, state, transport, and fate (Yates et al. 2021; Harrison, Sunday, and Rogers 2019), with variation in persistence due to factors such as temperature, pH, and microbial activity (Barnes and Turner 2016). Assuming that these dynamics also apply to nsDNA may lead to inappropriate conclusions being drawn about communities detected by natural samplers and, more importantly, ignore the potential to showcase different features afforded by utilizing alternative sample types. Macrobial eDNA varying from < 0.2 to > 180 μm has been reported in marine environments (Turner et al. 2014; Jo et al. 2019, 2020), suggesting that eDNA is available for capture in a range of states (e.g., cellular and extracellular) that likely differ in age, stability, and mobility (Jo and Minamoto 2021). Although the relationship between eDNA age and state is complex and unrealistic to study in full (Mauvisseau et al. 2022), it is generally accepted that “younger” DNA exists in larger particles and is more likely to be intramembranous (Jo 2023). Over time, larger particles are broken down and transported further from their source, impacting their detectability. As nsDNA is gaining attention, it is imperative to investigate which DNA states are and are not well represented by these samples to determine their viability for biomonitoring and the characteristics of the community data they provide (Harper et al. 2023; Brodnicke et al. 2023).

Here, we aim to assess the ability of two captive LMA sponges to capture eDNA in two different states and compare the uptake and persistence of nsDNA from sponge samples to aquatic eDNA from filtered water samples. First, we hypothesize that sponges will not effectively capture extracellular DNA due to its small size but will capture and accumulate larger (cellular) DNA particles. Second, we hypothesize that cellular DNA particles will persist longer as nsDNA than as eDNA in water samples, as DNA is captured and accumulates in sponge tissues. Our findings will provide an insight into whether communities detected by nsDNA have the potential to represent more temporally and spatially stable communities, relative to aquatic eDNA.

## 2 Materials and Methods

### 2.1 Facilities and Experimental Setup

This experiment was carried out at the Horniman Museum and Gardens (Horniman), London. Six independent aquaria were used, each consisting of a plastic 10 L tank and identical sump tank, containing a pump to maintain a flow of water between the tank and sump and primed bioballs (Maxspect Biosphere) for biological filtration. The total volume of water in the two tanks (main aquaria and sump) was 17.8 L at the start of the experiment. Aquaria were connected to an adjacent coral reef tank for 1 month prior to the experiment. This was done to establish the systems by seeding bioballs with microbes to establish biological filtration.

Sponges *Axinyssa* sp. and *Darwinella* sp. were used for the experiment. They grow naturally in the Horniman aquaria sumps, have different filtering characteristics, and have been used previously to investigate communities detected by nsDNA (Cai et al. 2022). Three weeks prior to the experiment, rocks with the encrusting sponges were trimmed using a band saw (gryphon) so the surface area of each sponge individual was roughly 50 cm^2^. One sponge individual of each species was placed in each tank 1 week prior to the start of the experiment for acclimatization. Tanks were isolated from the adjacent supply tank 3 days before the start of the experiment. Temperature and salinity of tanks were checked throughout the experiment and reverse osmosis water was added to correct for evaporation and maintain salinity at 35 ppt.

### 2.2 DNA Spiking

**FIGURE 1:**
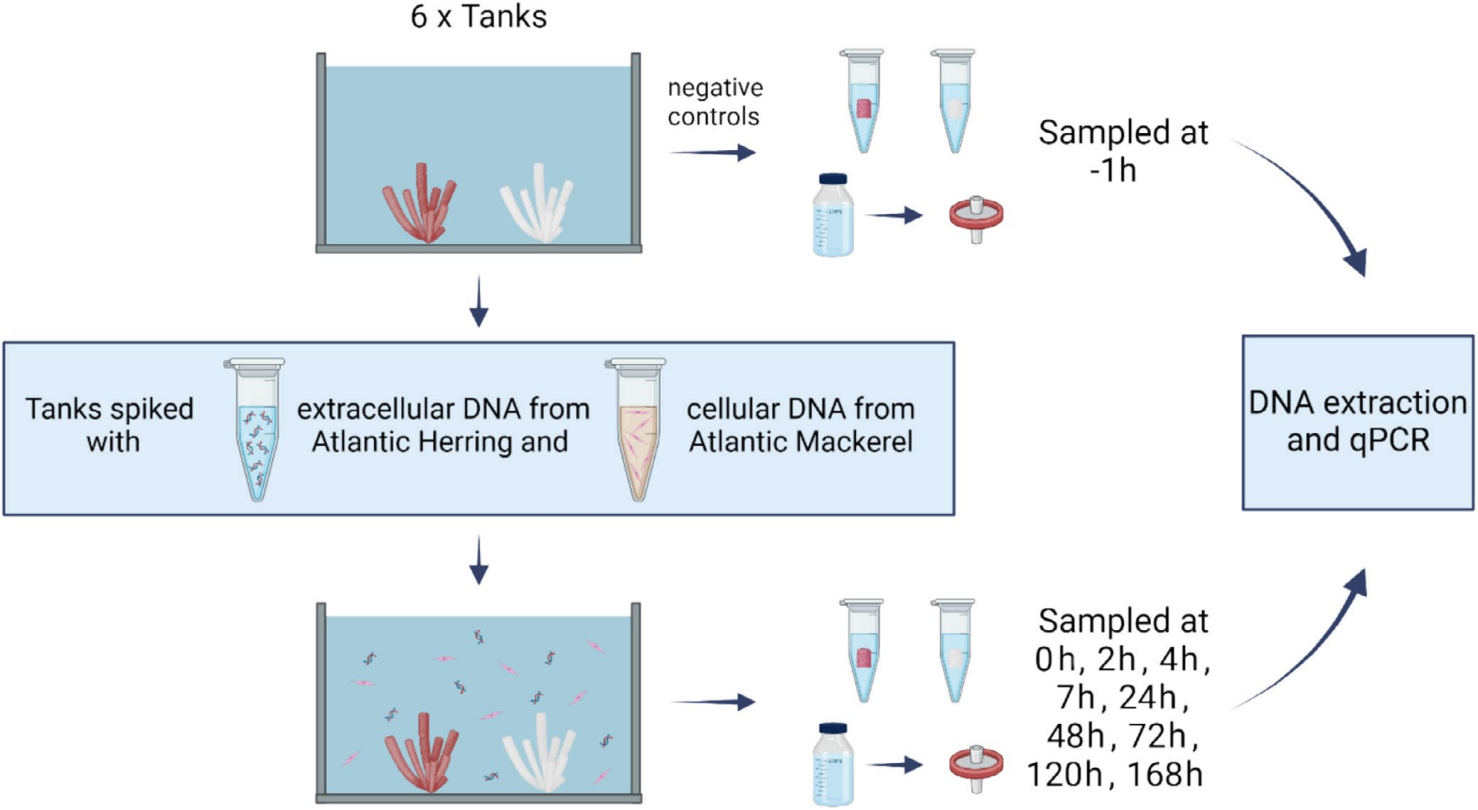
Overview of experimental setup and sampling regime. The workflow is depicted by arrows, including the samples collected at −1 h, the spiking event, and the samples collected over the remainder of the experiment. Graphic created using Biorender.

DNA from two fish species, Atlantic herring ( *Clupea harengus* ) and Atlantic mackerel ( *Scomber scombrus* ), was prepared for spiking the tanks. Extracellular DNA from Atlantic herring was prepared by extracting muscle tissue using the DNEasy Blood and Tissue kit (Qiagen; Hilden, Germany) following the manufacturer's protocol and quantified using a Qubit fluorometer. To create a source of cellular DNA, 5 g of muscle tissue from Atlantic mackerel was briefly homogenized in 50 mL of DNA‐free water using a sterilized hand blender. We acknowledge that this method may have caused the creation of some extracellular DNA due to the potential shearing effects; however, the blended muscle tissue still contained visible clumps of cells and is more representative of a cellular DNA source created naturally by mechanical forces. To determine the DNA concentration of the cellular source, DNA was extracted from 50 μL aliquots of the homogenized mackerel muscle using the DNeasy Blood and Tissue kit and quantified using a Qubit fluorometer. Each tank was spiked on April 19, 2023, with 7500 ng of extracted DNA from Atlantic herring (extracellular state) and 2 mL (equivalent to 4000 ng DNA) of blended muscle tissue from the Atlantic mackerel (cellular state). We chose to spike higher concentrations of the extracellular state DNA to ensure that it would be present at detectable levels while limiting the concentration of biological material (cellular DNA) to avoid overwhelming the natural biological filtration in the tank. See Figure 1 for experimental setup. Detectable concentrations of extracellular DNA were tested in a small gradient experiment prior to the primary experiment, with the final spiking volume chosen as it fell within ranges reported in the natural environment (Knudsen et al. 2019).

### 2.3 Sample Collection

Samples of tank water (eDNA) and sponge tissue (nsDNA) were collected over 7 days at −1, 0, 2, 4, 8, 24, 48, 72, 120, 168 h. The −1 h samples were collected prior to spiking the aquaria to confirm the absence of target DNA before the experiment. At each time point, one water sample and two sponge samples were collected from each tank (one per sponge species), totaling 180 samples over the course of the experiment. Elbow‐length sterile gloves were worn during sampling and changed between tanks and sponges to avoid any contamination. For eDNA samples, 250 mL of tank water was filtered directly from the tanks, using sterilized tubing and a vampire sampler handheld peristaltic pump (Bürkle; Germany), through a 0.22 μm PVDF Sterivex filter. After filtering, air was pushed through the Sterivex filter and they were capped and stored at −20°C. Sponge biopsies (1 cm^2^) were taken from the edge of the sponges using a sterile scalpel, placed in 2 mL cryovials with 1 mL of 100% ethanol, and stored at −20°C. Sampling blanks were collected each day by filtering 250 mL of reverse osmosis/deionized water. After the experimental period, the sponges were transferred to the Zoological Society of London and each sponge biopsy was transferred to a new 2 mL cryovial containing 100% ethanol in case the original ethanol was diluted with water from the sponges.

Sampling implements were sterilized prior to and disposed of after sample collection. Between sampling events, workspaces were decontaminated using 10% v/v bleach (6% sodium hypochlorite), and any non‐disposable sampling equipment was soaked in 10% v/v bleach for 30 min and thoroughly rinsed with reverse osmosis water between sampling events.

We note that all our sponges showed signs of regrowth at the sampling locations when they were checked 1 month after the experiment.

### 2.4 Laboratory Procedures

#### 2.4.1 Extraction Trials and Inhibition Testing

Extra sponge samples from the Horniman were collected, as described above, for method validation. Samples of both sponge species were dabbed dry to remove excess ethanol and 0.025 g was placed in a clean tube for extraction. To assess for successful co‐extraction, optimal primer/probe concentrations, and potential inhibition, extracted DNA from herring and mackerel (10 ng each) was added to the tubes containing sponge biopsies prior to addition of lysis buffer. All samples were extracted using the DNeasy Blood and Tissue kit and were incubated at 56°C overnight prior to extraction following the kit protocol. Concentrations of extracted DNA were measured using a Qubit fluorometer with high‐sensitivity chemistry. PCR inhibition was observed in both sponge species.

We assessed multiple methods to reduce PCR inhibition (see Quantitative PCR of eDNA and nsDNA samples section below for PCR protocol). The samples of co‐extracted sponge and fish DNA were treated with either the OneStep PCR inhibitor removal kit (ZYMO; California, USA) or DNeasy PowerClean Pro Cleanup kits (Qiagen; Hilden, Germany). We also ran dilution series, with dilutions of 1:10, 1:20, 1:50, and 1:100, and included bovine serum album (BSA) (0.5 ng/μL) in replicate qPCR reactions. Due to inconsistent removal of inhibitors using these methods, we also trialed co‐extracting sponge and target DNA (starting material same as described above) using Qiagen PowerSoil Pro kit and CTAB extractions. Again, with these extracts, we ran a dilution series and repeated PCR assays (as above). See Appendix S1 for further details and outcomes of method validation tests.

#### 2.4.2 Experimental Extractions

Water samples were extracted using the DNeasy Blood and Tissue kit following the modified protocol in Spens et al. (2017), including an overnight incubation at 56°C. To reduce PCR inhibition, sponge samples were extracted using the PowerSoil Pro kit, following the manufacturer's protocol (see extraction trials above). Prior to extraction of the sponge samples, excess ethanol was blotted off using sterile filter paper as recommended by Harper et al. (2023), and a standardized weight (0.03 g) of sponge tissue was finely cut with sterilized scissors and placed into a clean tube. Extraction blanks were included during each extraction and DNA concentration was measured using a Qubit fluorometer with high sensitivity chemistry.

#### 2.4.3 Quantitative PCR of eDNA and nsDNA Samples

Quantitative PCR (qPCR) assays targeting these two species were designed by (Knudsen et al. 2019) and were used to track the two different states of DNA throughout the experiment. Details of the primers can be found in Table 1. Both target a short fragment (85–100 bp) of the mitochondrial cytochrome b subunit and muscle tissue from both species was available as commercial food products to create the two DNA states.

**TABLE 1:** Details of the assays used for the detection of Atlantic mackerel and herring (Knudsen et al. 2019).

Target species	Assay name	Sequence (5′‐3′)	Target fragment (bp)
Atlantic mackerel ( *Scomber scombrus* )	Scosco_CYBF14517	TTCCCTGCTTGGTCTCTGTT	100
	Scosco_CYBR14597	GGCGACTGAGTTGAATGCTG	
	Scosco_CYBP14541	FAM‐TTCCCAAATCCTCACAGGACTATTC‐BHQ1	
Herring ( *Clupea harengus* )	Cluhar_CYBF14928	CCCATTTGTGATTGCAGGGG	86
	Cluhar_CYBR15013	CTGAGTTAAGTCCTGCCGGG	
	Cluhar_CYBP14949	FAM‐TACTATTCTCCACCTTCTGTTCCTC‐BHQ1	

Standard curves were calculated for each assay using a dilution series of double‐stranded DNA (Eurofins) from Atlantic mackerel and herring in tRNA + TE stabilization buffer. All work with concentrated synthetic DNA (> 50 copies/μL) was carried out in a room separate from eDNA processing. A dilution series from 1.0 × 10^10^ copies/μL to 1.6 × 10^−2^ copies/μL was run for each assay to determine the limit of detection (LOD) and limit of quantification (LOQ). We performed 20 technical replicates for concentrations less than 10 copies/μL, and 8 technical replicates for higher concentration dilutions.

Both assays were run on nsDNA and eDNA samples to detect and quantify the extracellular and cellular spiked DNA sources. Five qPCR replicates were run for each sample with both assays. Each reaction had a total volume of 20 μL, comprising 10 μL of TaqMan Universal PCR Master Mix (Life Technologies), 5 μL ddH_2_O, 1 μL of each primer (forward and reverse) (10 μM each), 1 μL of probe (2.5 μM), and 2 μL of template DNA. A 1:10 dilution of sponge DNA extract was used to reduce PCR inhibition. The qPCR settings were an initial preheat at 50°C for 5 min, 10 min at 95°C, followed by 50 cycles at 95°C for 30 s and 60°C for 1 min. Two positive controls and nontemplate negative controls were run on each plate. qPCR reactions were run on a StepOnePlus Real‐Time PCR System (Life Technologies).

### 2.5 Analysis

All statistical analysis was completed in R (R Core Team 2022). The LOD and LOQ were calculated using the eLowQuant script (Lesperance et al. 2021). Values above the LOD for respective assays were considered a positive detection in the analysis of detection rate but only detections above the LOQ were used for quantitative analysis of DNA copy number. For these analyses, all replicates with values below the LOD, LOQ, and non‐detects were assigned a cycle threshold value (*C*_t_) 0.5 above their respective assay's LOD to minimize bias in the analysis.

#### 2.5.1 DNA Concentrations

Linear models of the standard dilution series for each assay were used to calculate the number of copies per reaction. Copies for each sample (100 μL of eDNA or nsDNA extract) were calculated by multiplying by 50 for eDNA samples and by 500 for sponge samples, to account for the 1:10 dilution used in sponge DNA qPCR reactions. For analysis at the sample level, this was calculated using the average *C*_t_ score across the 5 technical replicates.

A generalized linear model (GLM) analysis was performed to evaluate the effect of sample type (eDNA or nsDNA), DNA state (extracellular or cellular), time, and potential interactions on DNA copies per sample. DNA copies per sample were log‐transformed for normalization after visual inspection and the model had an inverse gamma distribution as the data was left‐skewed and continuous. The full model formula was glm (log (DNA copies) ~ sample type + DNA state + sample type*DNA state + time + time*sample type). Initially, a general linear mixed model (GLMM) was used but tank as a random effect was removed as minimal variation in DNA concentrations between tanks resulted in singularity issues during model fitting and the reversion to a GLM.

For both analyses, AIC was used to assess the best models and the DHARMa package (Hartig 2022) was used to assess model suitability and fit including distribution of residuals, uniformity, and zero inflation.

#### 2.5.2 Detection Rate

Detection rate per sample was calculated by dividing the number of positive qPCR reactions by the number of technical replicates (5). A detection rate of 1 indicates that all technical replicates were successful (100%), while a detection rate of 0 indicates that there were no positive detections (0%).

A GLMM, using the glmmTMB package in R (Brooks et al. 2017), was used to evaluate the effect of sample type, DNA state, time, and potential interactions on detection rate. The model used a zero‐inflated binomial distribution, with the explanatory variables sample type, DNA state, time, and the interactions between type and state and time and type. Tank was included as a random effect and time was log‐transformed for data normalization. The full model formula was as follows; glmm (detection rate ~ sample type + DNA state + sample type*DNA state + log(time) + log(time)*sample type + tank (random effect)).

*DNA persistence*.

Mann–Whitney tests were performed to test for significant differences in DNA persistence, testing DNA copies per sample (log transformed) from the 120‐ and 168‐h samples (combined) against sample type for both DNA states.

## 3 Results

### 3.1 Extraction Trials and Inhibition Testing

Extraction trials found there to be a high degree of PCR inhibition when co‐extracting target DNA with sponge tissue. Target DNA was reliably detected across technical replicates in *Darwinella* sp. samples using the DNeasy Blood and Tissue kit followed by either a 1:20 dilution of the DNA extract, or PCR inhibitor removal via PowerClean Pro clean up kit. Target DNA was also reliably amplified in *Darwinella* sp. samples extracted with the PowerSoil Pro kit and diluted to 1:10. Target DNA co‐extracted with *Axinyssa* sp. samples did not consistently amplify when extracted using the DNeasy Blood and Tissue kit, followed by dilution or after inhibitor removal with the OneStep or the PowerClean Pro Cleanup kit (Appendix S1). CTAB extractions were also unsuccessful for both sponge types. In samples extracted with the PowerSoil Pro kit, which includes an inhibitor removal step, it was possible to amplify target DNA co‐extracted with *Axinyssa* sp. with dilution. However, a dilution of 1:50 or higher was required for target DNA to be detectable when co‐extracted with *Axinyssa* sp., while only a 1:10 dilution was required for consistent results in *Darwinella* sp. Trialing this extraction method and dilution on experimental *Axinyssa* samples, DNA spiked into the tanks was too low to be detected, even at initial timepoints when target DNA concentrations were likely to have been at their highest in the sponge tissue. As such the *Axinyssa* sp. samples were omitted from downstream analysis, and the PowerSoil Pro kit was taken forward as the most reliable extraction method for *Darwinella sp*. samples.

### 3.2 Experimental Extractions

As a result, a total of 60 eDNA samples and 60 nsDNA (*Darwinella* sp.) samples were extracted and used in this analysis. A total of 1200 qPCR replicates were run, consisting of 600 replicates of both herring and mackerel assays. There was no amplification detected for either assay in the −1 h samples, sampling, or extraction blanks indicating that target DNA was absent prior to the spiking event and that there was no contamination during sampling or extractions. There was also no amplification in the negative controls included on all qPCR plates.

### 3.3 Limits of Detection and Quantification

For the cellular, Atlantic mackerel assay, the LOD and LOQ were 0.15 copies/μL. For the extracellular, herring assay, the LOD was 0.05 copies/μL and the LOQ was 0.1 copies/μL. The *R* ^2^ and amplification efficiency for the mackerel assay were 0.9925% and 96.41%, and 0.9995% and 98.05% for the herring assay. In total 684/1200 reactions were below the LOD, with a further 15 lower than the LOQ but above the LOD.

### 3.4 DNA Concentrations

**FIGURE 2:**
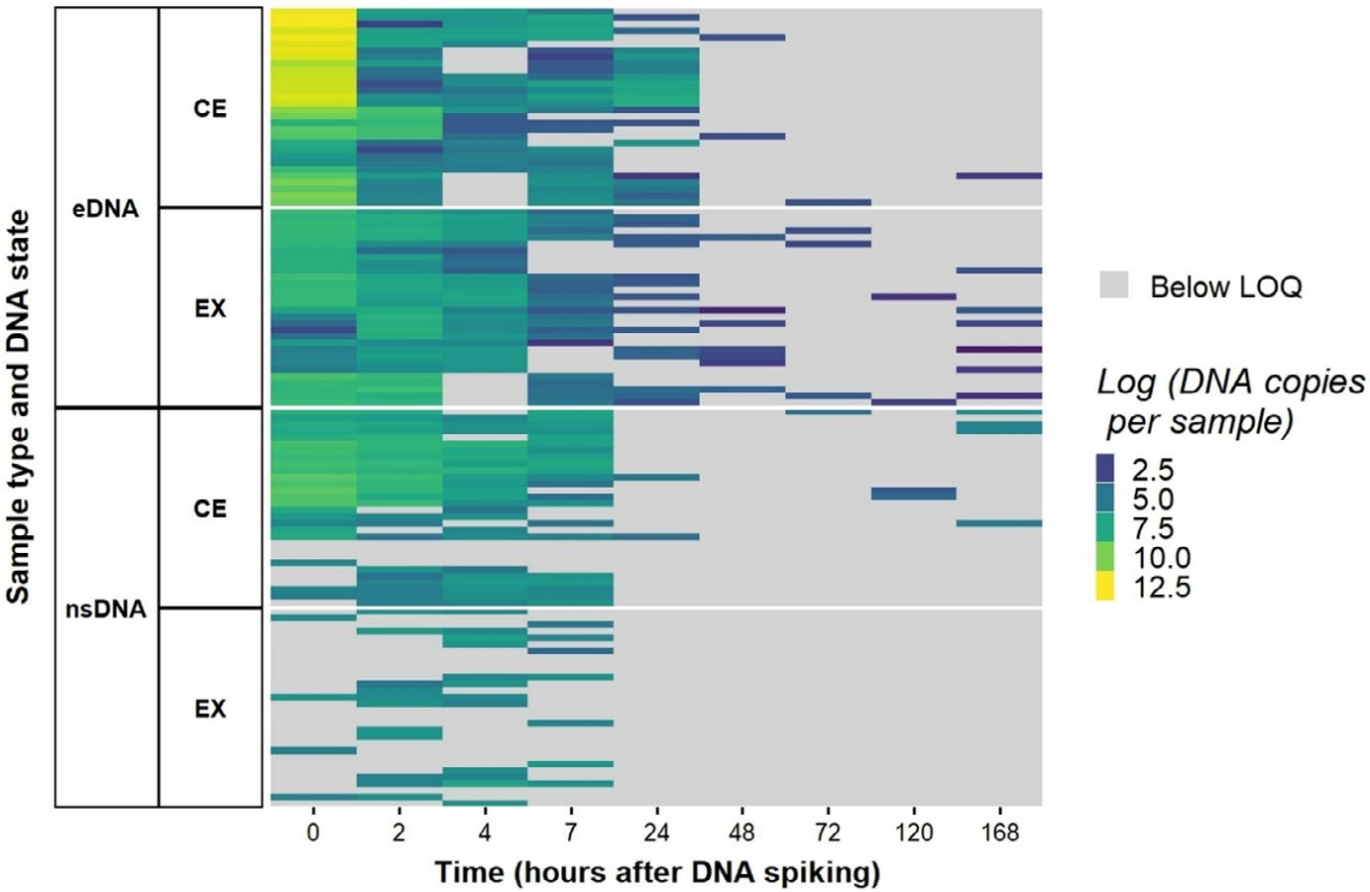
Heatmap of DNA copies per sample (log transformed) of every technical replicate for each sample during the experimental period. Labels on the *y*‐axis show sample type (eDNA and nsDNA) and DNA states, extracellular (EX) and cellular (CE). Time (hrs) since the spiking event is shown in chronological order on the *x*‐axis.

Sample type was found to have a significant effect on DNA copies per sample, with fewer DNA copies per sample observed in nsDNA samples compared to eDNA samples. There was no significant effect of DNA state although there was a significant interaction effect, with significantly fewer copies of extracellular DNA detected in nsDNA samples. There was also a significant interaction between DNA copies per sample over time, with higher concentrations detected at early time points in eDNA samples than in nsDNA samples (Figure 2). Model results can be seen in Table 2.

**TABLE 2:** Results of the generalized mixed effects models run to evaluate the effects of sample type, DNA state, and time on detection rate and log (copies per sample).

Predictors	Detection rate			Log (DNA copies per sample)		
	Odds ratios	CI	*p*	Estimates	CI	*p*
Intercept	246.56	83.11–731.52	**< 0.001**	1.15	1.12–1.18	**< 0.001**
Type [nsDNA]	0.03	0.01–0.11	**< 0.001**	1.16	1.10–1.23	**< 0.001**
DNA state [EX]	1.63	0.91–2.92	0.098	1.02	0.98–1.07	0.246
Log(time)	0.19	0.14–0.25	**< 0.001**			
Type [nsDNA] × DNA state [EX]	0.08	0.04–0.19	**< 0.001**	1.34	1.21–1.50	**< 0.001**
Type [nsDNA] × log (time)	1.98	1.41–2.78	**< 0.001**			
Time				1.01	1.01–1.01	**< 0.001**
Type [nsDNA] × time				1.00	1.00–1.00	**0.012**
Zero‐inflated model						
Intercept	0.06	0.02–0.18	**< 0.001**			
Random effects						
*σ* ^2^	3.29					
τ_00_	0.26_tank_					
ICC	0.07					
N	6_tank_					
Observations	216			216		
Marginal *R* ^2^/Conditional *R* ^2^	0.685/0.708			0.787		

*Note:* Text in bold indicates *p* values < 0.05.

### 3.5 Detection Rate

**FIGURE 3:**
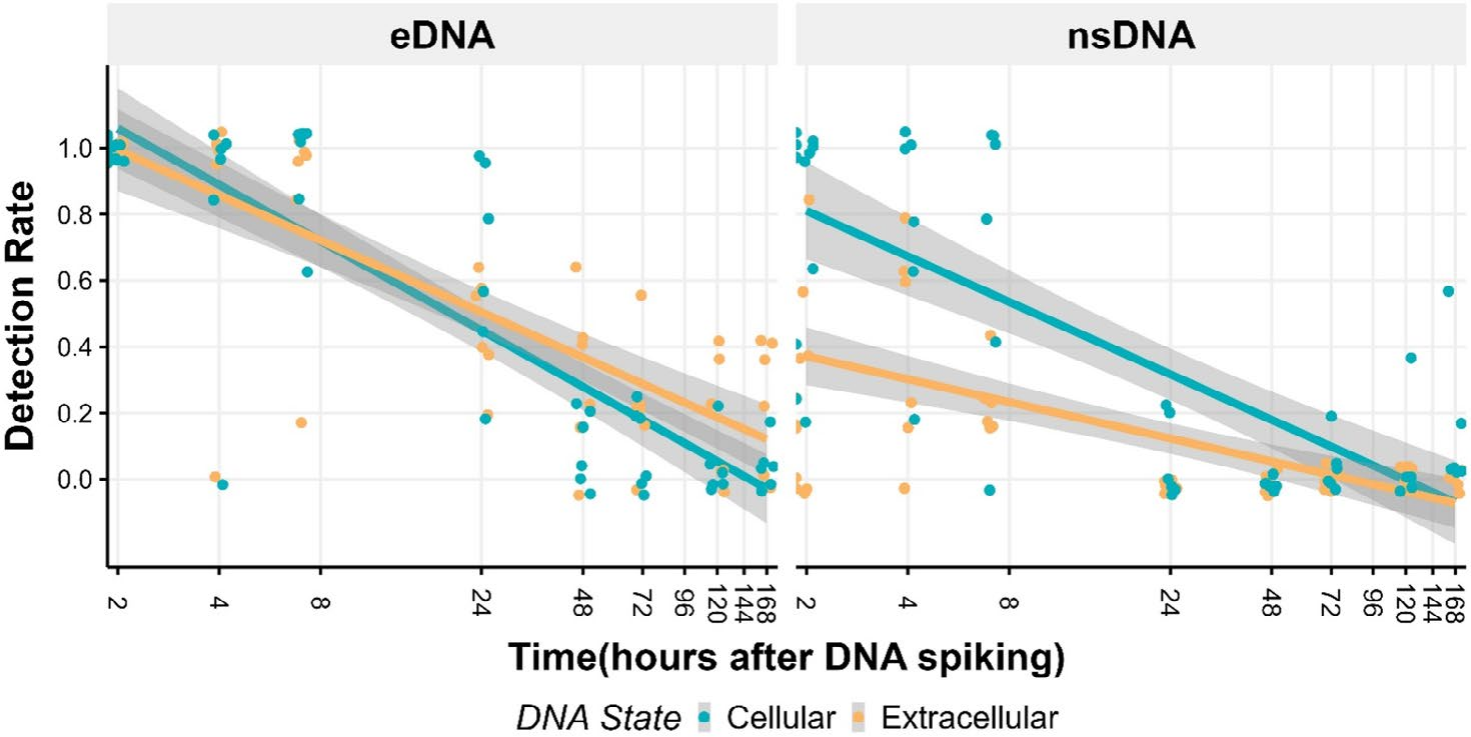
Linear changes in detection rate, across 5 technical replicates, over time for both cellular and extracellular DNA. in eDNA and nsDNA samples. Points have been jittered on the *x*‐ and *y*‐axes for improved visualization.

Sample type was found to also have a significant effect on detection rate, with nsDNA samples having a lower detection rate than eDNA samples. Again, there was also a significant interaction between sample type and DNA state with lower detection rates of extracellular DNA in nsDNA samples (Figure 3). There was also a significant interaction between sample type and time, with much higher initial detectability in eDNA samples than nsDNA samples (Figure 3 and Table 2).

### 3.6 DNA Persistence

Mann–Whitney tests found that the DNA copies per sample (*W* = 108, *p* = 0.007) and detection rate (*W* = 114, *p* = 0.0027) of extracellular DNA in the 140‐ and 168‐h sampling events was significantly higher in eDNA than nsDNA. There was no significant difference in the persistence of cellular DNA between the two sample types.

## 4 Discussion

Here, we show that although marine sponges do act as natural samplers of DNA found in the environment, DNA concentrations and detectability differ from that captured by aquatic eDNA samples, and that extracellular DNA is captured poorly by marine sponges. Detection of cellular DNA in the nsDNA samples was found to be comparable to that found in aquatic eDNA, although concentrations in eDNA samples immediately after spiking were understandably magnitudes higher. Overall, we confirm the usability of sponges as natural samplers of eDNA and provide insight into the potential origin of communities reflected by the metabarcoding of spongy tissues.

There was significantly lower detection and lower concentrations of extracellular DNA found in nsDNA compared to eDNA samples. There were some positive detections in most samples taken within 7 h of spiking the mesocosms; however, there was no detection of extracellular DNA in nsDNA samples beyond the 1st day. This indicates that DNA in an extracellular state is either poorly captured by this natural sampler and/or is rapidly broken down within sponge tissues, potentially by microbes living within the organisms (Brodnicke et al. 2023; Zulkefli, Kim, and Hwang 2019; Moitinho‐Silva et al. 2017). In contrast, cellular DNA was more readily detectable in the nsDNA samples, and at higher copy numbers than extracellular DNA over the experimental period. However, the detection of cellular DNA did also drop off in nsDNA samples after the 7‐h sampling point, compared to after 24 h in the eDNA samples. Due to PCR inhibition observed in nsDNA samples in our extraction trials, these reactions were run with a 1:10 dilution, which likely reduced the sensitivity of the assay and, therefore, the detection rate in these samples compared to eDNA samples. Inhibition has been previously noted in other nsDNA studies (Harper et al. 2023; Turon, Antich, et al. 2020; Cai et al. 2022) and is expected due to the high prevalence of bioactive metabolites and humic compounds that can be found in sponges (Vargas et al. 2012; Schrader et al. 2012). The necessary differences in extraction method (both in terms of extraction kit and quantity of sample processed) and dilutions required between eDNA and nsDNA samples align with how these sample types would be processed in nonexperimental settings and, importantly, highlight the methodological challenges associated with nsDNA sampling. As dilutions are commonly required to remove inhibition from nsDNA samples, future work needs to determine how this impacts the detectability of rare taxa in community metabarcoding studies and if increased PCR replicates could moderate this effect.

Our finding that extracellular DNA was picked up poorly in nsDNA samples indicates that metabarcoding of sponges collected in situ may describe communities originating from a subset of the extra‐organismal DNA available in the environment. Environmental DNA is likely released by organisms as larger cellular particles that break down over time and space from its origin (Harrison, Sunday, and Rogers 2019; Joseph et al. 2022), leading to a common concern in aquatic eDNA of false positives caused by transport of eDNA (Burian et al. 2021). Our findings suggest that DNA represented in nsDNA samples is more likely to originate from sources closer to the sampling location, as it is yet to have had the time to breakdown and be transported to large distances, potentially leading to a better representation of more local organisms. Of the cellular DNA concentrations detected between 0 and 7 h after spiking, nsDNA samples also provide a more stable signal than corresponding eDNA samples in terms of copy number. This may indicate that natural samplers could be less impacted by natural spiking events, such as predation, spawning, or mortality events, which can produce large quantities of eDNA and dominate aquatic eDNA samples (Ip et al. 2022) and be less likely to reflect small scale temporal signals due to daily changes in organism behavior and movement (Dowell et al. 2024). However, our findings reflect a small‐scale, controlled mesocosm experiment utilizing limited sponge species and results will likely differ in natural systems and between sponge taxa. As sponge species are not globally distributed and it cannot be guaranteed that particular sponge species will be found in every ecosystem of interest, the use of a particular species as a global natural sampler is not possible. As such, expanding experiments like this to in situ environments and comparing nsDNA profiles across sponge species, orders, and families is an important next step in the uptake of sponges as nsDNA tools.

Persistence of cellular DNA was found to be similar across eDNA and nsDNA samples, with low detection probabilities found in both sample types after 1 day. However, if we consider cellular DNA detections across only the last two sampling points (120 and 168 h), with 6 replicate tanks and 5 qPCR replicates, there were a total of 60 detection opportunities. We received only 1/60 positive detection in eDNA samples compared to 7/60 positive detections in nsDNA samples, providing some evidence for increased persistence of cellular DNA in nsDNA samples. This may be due to the potential accumulation of DNA in the sponge tissues; however, we would not expect the low detection probabilities seen at 48 and 72‐h. This result could reflect the sponges removing cellular DNA from the water in each enclosed tank system in small quantities throughout the experiment. Sporadic detection in nsDNA samples toward the end of the experiment could also indicate that DNA distribution within sponge tissues is not homogenous and that larger or replicate samples may be necessary to obtain a more comprehensive picture of diversity from in situ samples of this type, aligning with common recommendations for aquatic samples (Harper et al. 2023). The persistence of extracellular DNA beyond that of cellular DNA in the eDNA samples is an unexpected result. This could be due to the lack of uptake of extracellular DNA by sponge tissues (compared to cellular DNA) and potentially due to greater initial concentrations of extracellular DNA (7500 ng) spiked into the tanks compared to cellular DNA (4000 ng). The latter was a methodological choice to ensure the extracellular DNA was detectable while avoiding overwhelming the natural biological filtration in the tank with the addition of too much biological material (cellular DNA). However, overall, these results and existing evidence of DNA persistence in sponge tissues (Cai et al. 2022) highlight the use of nsDNA in detecting more stable DNA states than aquatic samples, and the need for further work to define the size of the sample and replicates needed to provide an accurate picture of local communities from natural samplers.

Here, the combination of the PowerSoil extraction kit, which includes inhibitor removal steps, and dilution of DNA extracts did allow for successful target DNA amplification in both sponge types. However, dilutions of 1:100 were required to reliably detect fish DNA co‐extracted with *Axinyssa* sp. tissue. This made it impractical to use this sponge for the quantification of low concentrations of DNA in this experiment. This sponge has previously been used to successfully describe mock communities via metabarcoding in the same facilities, showing similar persistence patterns to eDNA samples (Cai et al. 2022). Our study represents the first account of using nsDNA for species‐specific qPCR for macrobial targets, which enabled targeted testing for PCR inhibition with this sample type. It is possible that sponge‐derived inhibitors were qPCR‐specific, interfering with the fluorescence of probes in the reaction. However, this was not found to be an issue in previous studies using extracts from a different sponge species for probe‐based qPCR investigating microbial symbionts (Cassler et al. 2008). There are a variety of methods that could alleviate the inhibition observed in this study. Further recommendations would be to trial a different master mix (e.g., TaqMan Environmental MasterMix) or transition to other platforms, such as dPCR and ddPCR (Cao, Raith, and Griffith 2015), this study does highlight a degree of inhibition observed in nsDNA samples which may not be easily detected in metabarcoding methods. This warrants further investigation, as suggested above, to ensure appropriate sponge types are used and the potential biases of universal primers and inhibition in nsDNA are understood.

Our study provides important insights into the likely origin of communities described by sequencing of nsDNA samples, as coming from larger, cellular eDNA rather than extracellular DNA. We provide further evidence for the potential of nsDNA samples for species detection but caution that little is understood about how and why these samples differ from established eDNA methods. Inhibition of PCRs highlights that the selection of suitable candidates for nsDNA samples requires more attention. There are many factors, including sponge morphology, pumping rates, and microbial abundance, which may bias not only DNA capture and persistence mechanisms but also the molecular methods required to extract community DNA from nsDNA samples. However, with increasing understanding and optimization of nsDNA methods, nsDNA looks to provide an opportunity to utilize new and archived sponge samples, tapping into an extra source of biodiversity data.

## Author Contributions

**Rosalie Dowell:** conceptualization (lead), data curation (lead), formal analysis (lead), funding acquisition (equal), investigation (lead), methodology (lead), visualization (lead), writing – original draft (lead). **Jamie Craggs:** conceptualization (supporting), methodology (supporting), project administration (equal), resources (lead), supervision (equal), writing – review and editing (equal). **Catherine Head:** conceptualization (supporting), funding acquisition (equal), supervision (equal), writing – review and editing (equal). **Chris Yesson:** conceptualization (supporting), funding acquisition (equal), supervision (equal), writing – review and editing (equal). **Emma Ransome:** conceptualization (equal), funding acquisition (equal), supervision (lead), writing – review and editing (equal).

## Conflicts of Interest

The authors declare no conflicts of interest.

## Supporting information


Appendix S1.

## Data Availability

The data that supports the findings of this study are available in the Supporting Information of this article.
